# Quantum Information Entropy for Another Class of New Proposed Hyperbolic Potentials

**DOI:** 10.3390/e25091296

**Published:** 2023-09-05

**Authors:** R. Santana-Carrillo, Roberto de J. León-Montiel, Guo-Hua Sun, Shi-Hai Dong

**Affiliations:** 1 Centro de Investigación en Computación, Instituto Politécnico Nacional, UPALM, Mexico City 07738, Mexico; 2 Instituto de Ciencias Nucleares, Universidad Nacional Autónoma de México, Apartado Postal 70-543, Mexico City 04510, Mexico; 3 Research Center for Quantum Physics, Huzhou University, Huzhou 313000, China

**Keywords:** Shannon entropy, hyperbolic potentials, single well potential, double well potential, BBM inequality, Fisher entropy

## Abstract

In this work, we investigate the Shannon entropy of four recently proposed hyperbolic potentials through studying position and momentum entropies. Our analysis reveals that the wave functions of the single-well potentials $U_{0,3}$ exhibit greater localization compared to the double-well potentials $U_{1,2}$. This difference in localization arises from the depths of the single- and double-well potentials. Specifically, we observe that the position entropy density shows higher localization for the single-well potentials, while their momentum probability density becomes more delocalized. Conversely, the double-well potentials demonstrate the opposite behavior, with position entropy density being less localized and momentum probability density showing increased localization. Notably, our study also involves examining the Bialynicki–Birula and Mycielski (BBM) inequality, where we find that the Shannon entropies still satisfy this inequality for varying depths $\bar{u}$. An intriguing observation is that the sum of position and momentum entropies increases with the variable $\bar{u}$ for potentials $U_{1,2,3}$, while for $U_{0}$, the sum decreases with $\bar{u}$. Additionally, the sum of the cases $U_{0}$ and $U_{3}$ almost remains constant within the relative value 0.01 as $\bar{u}$ increases. Our study provides valuable insights into the Shannon entropy behavior for these hyperbolic potentials, shedding light on their localization characteristics and their relation to the potential depths. Finally, we extend our analysis to the Fisher entropy ${\bar{F}}_{x}$ and find that it increases with the depth $\bar{u}$ of the potential wells but ${\bar{F}}_{p}$ decreases with the depth.

## 1. Introduction

Shannon entropy, named after Claude Shannon, is a fundamental concept in information theory and holds immense importance across various fields, including computer science, communication, data compression, cryptography, machine learning, and more [1,2,3,4,5,6,7,8,9,10,11,12,13,14,15,16,17,18,19,20,21,22,23,24,25,26,27,28,29,30,31,32]. This concept stems from the notion of quantifying uncertainty or randomness within an information source or dataset.

There are several key reasons why Shannon entropy is essential. Firstly, it serves as a measure of information content [1,2,3,4,5,6]. When applied to a random variable or data distribution, Shannon entropy quantifies the amount of information present. High entropy indicates greater uncertainty and more information, while low entropy signifies predictability and less information. Secondly, Shannon entropy plays a crucial role in data compression techniques [33]. These methods aim to reduce the number of bits required to represent information while preserving its essential characteristics. The theoretical lower bound for lossless data compression is given by Shannon entropy, suggesting that optimal compression should strive to achieve or even surpass this entropy level in bits per symbol. Furthermore, in communication systems, Shannon entropy helps determine the minimum average number of bits needed to transmit information reliably from sender to receiver [1]. It aids in designing efficient coding schemes that can withstand noise and interference in communication channels. Additionally, Shannon entropy finds applications in data analysis and pattern recognition [34]. For instance, it is used in decision trees and feature selection algorithms to measure dataset homogeneity and determine the best features for splitting. In the context of machine learning [34], Shannon entropy is vital for calculating information gain when constructing decision trees. Information gain helps identify the most informative features for data splitting, leading to improved classification performance. The significance of Shannon entropy extends to security and cryptography [35]. In this realm, entropy is used to evaluate the strength of encryption keys and random number generators. Higher entropy in cryptographic keys ensures better protection against brute-force attacks. Moreover, entropy is instrumental in data quality assessment and anomaly detection. It can be utilized to identify abnormal patterns or outliers in datasets, as unusual data points tend to exhibit lower probabilities and result in higher entropy values. In summary, Shannon entropy provides a rigorous and quantifiable measure of uncertainty and information content, making it a fundamental tool for understanding, modeling, and optimizing various systems and processes involving information. Its applications span numerous domains and remain relevant in an era characterized by ever-expanding data and information-driven technologies.

**Figure 1:**
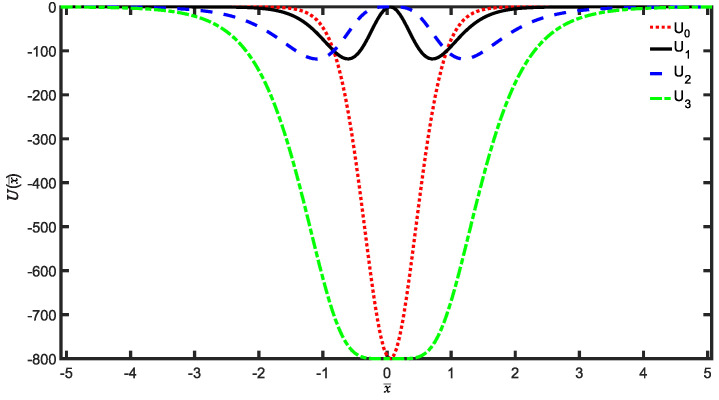
Plot of potentials $U_{i}\left(i=0,1,2,3\right)$ as a function of variable $\bar{x}$.

In this work, we are mainly concerned with the uncertainty and information content of quantum systems through a measure of quantum information entropy. Up to now, the study of Shannon information entropy has been extended to encompass many quantum soluble potentials. These potentials are amenable to exact determination of probabilities due to their solvability in quantum states. Consequently, the probabilities associated with quantum states can be used in a manner analogous to classical probabilities in Shannon’s entropy formula, facilitating the definition of Shannon entropy for quantum states. Among the soluble potentials, the hyperbolic potentials have played a vital role in semiconductor physics, including research related to graphene [14,36,37,38,39]. Recently, we have explored quantum information entropy for hyperbolic potentials $U_{1,2}$ within the time-independent traditional and fractional Schrödinger equation [29,40,41]. These hyperbolic potential wells, being single-well or double-well potentials were studied by Wang et al. [42]. It should be recognized that the denominator of these potentials proposed in Ref. [42] is $\cosh^{4}\left(\bar{x}\right)$ type. Recently, we have extended this kind of hyperbolic potentials to a more complicated hyperbolic potentials though replacing this denominator by $\cosh^{6}\left(\bar{x}\right)$ [43] as shown in Figure 1. We see that the minimum values of the single-well potentials $U_{0,3}$ are −u but those of the double-well potentials $U_{1,2}$ are −4u/27, which are located at ${\bar{x}}_{1_{min}}=\pm arc\cosh\left(\sqrt{3/2}\right)$ for $U_{1}$ and ${\bar{x}}_{2_{min}}=\pm arc\cosh\left(\sqrt{3}\right)$ for $U_{2}$, respectively.

(1) $$U_{q}\left(x;u\right)=\left\{\begin{array}{cc} -\alpha \frac{\bar{u}}{\cosh^{6}\left(\bar{x}\right)}, & q=0,\\ -\alpha \frac{\bar{u}\sinh^{2}\left(\bar{x}\right)}{\cosh^{6}\left(\bar{x}\right)}, & q=1,\\ -\alpha \frac{\bar{u}\sinh^{4}\left(\bar{x}\right)}{\cosh^{6}\left(\bar{x}\right)}, & q=2,\\ -\alpha \bar{u}\left[1-\tanh^{6}\left(\bar{x}\right)\right], & q=3. \end{array}\right.$$

where $\alpha =\frac{\hbar^{2}}{2m\;L^{2}}$ and dimensionless position $\bar{x}=x/L$ and $\bar{u}=\frac{2mL^{2}}{\hbar^{2}}u$. Here, the length *L* describes the steepness of the potential and *u* shows its depth.

This work is structured as follows. In Section 2, we introduce the essential principles of quantum information entropy. We establish the theoretical foundation and the necessary mathematical framework for the subsequent analyses. In Section 3, we present the outcomes of our study. Specifically, we provide detailed examinations of the wave functions corresponding to hyperbolic potential wells. Furthermore, we investigate the position and momentum entropies densities, along with the position ${\bar{S}}_{x}$ and momentum ${\bar{S}}_{p}$ Shannon entropies, while varying the depth $\bar{u}$ of the potential wells. Additionally, we explore the characteristics of higher excited states except for the ground, first, second, and third states. For instance, we discuss the 15th excited states in the context of single-well potentials $U_{0,3}$, while focusing on the 12th and 4th excited states for double-well potentials $U_{1,2}$. This distinction is relevant to the number of bound states permitted in the systems under investigation. Moreover, we perform a comparative analysis by studying the Fisher entropy in relation to the Shannon entropy. In the final Section 4, we summarize the key findings and conclusions drawn from our research.

## 2. Fundamental Concepts

We start with the one-dimensional time-independent Schrödinger equation

(2) $$\left(-\frac{\hbar^{2}}{2m}\frac{d^{2}}{dx^{2}}+V\left(x\right)\right)\varphi \left(x\right)=E\varphi \left(x\right),$$

where the geometrical confining potential V(x) can take any of the forms given in Equation (1). In our recent work [43], we successfully tackled the challenge of finding exact solutions for this system. These solutions were determined to be represented by the confluent Heun function. However, a drawback arose due to the involvement of the energy spectrum parameter *E* in this function. As a consequence, the wave function in momentum space could not be readily obtained during the Fourier transformation of the wave function. As a result of this limitation, we are now prepared to explore quantum information entropy through numerical methods. The method used here is named the Finite Difference Method to solve the Schrödinger equation [44]. Using the second order centered derivative formula, we are able to discretize Equation (2) as follows

(3) $$-\frac{\hbar^{2}}{2m}\left(\frac{\varphi_{i+1}-2\varphi_{i}+\varphi_{i-1}}{s^{2}}\right)+V_{i}\varphi_{i}=E\varphi_{i},$$

where *s* is the step size. Let us suppose we are going to solve this equation in the region x∈[a,b], then we can create N+1 grid points such that $x_{0}=a$ and $x_{N}=b$. Since the particle is confined in the region x∈[a,b], this results in the boundary conditions $\varphi_{0}=0$ and $\varphi_{N}=0$. Thus, we only need to compute $\varphi_{i}$ for the remaining N−1 grid points, that is i=1,2,3,…,N−1. The Hamiltonian can be written as

(4) $$\left[\begin{array}{cccccc} 2\tau_{0}+V\left(x_{1}\right) & -\tau_{0} & 0 & 0 & 0 & 0\\ -\tau_{0} & 2\tau_{0}+V\left(x_{2}\right) & -\tau_{0} & 0 & 0 & 0\\ 0 & -\tau_{0} & \ddots & \ddots & 0 & 0\\ 0 & 0 & \ddots & \ddots & \ddots & 0\\ 0 & 0 & 0 & \ddots & \ddots & -\tau_{0}\\ 0 & 0 & 0 & 0 & -\tau_{0} & 2\tau_{0}+V\left(x_{N}\right) \end{array}\right]\left[\begin{array}{c} \varphi_{1}\\ \varphi_{2}\\ \vdots \\ \vdots \\ \varphi_{N} \end{array}\right]=E\left[\begin{array}{c} \varphi_{1}\\ \varphi_{2}\\ \vdots \\ \vdots \\ \varphi_{N} \end{array}\right],$$

where $\tau_{0}=\frac{\hbar^{2}}{2ms^{2}}$. The reader can refer to [44] for more detailed information. In particular, the reader are also able to find the Matlab code there.

**Figure 2:**
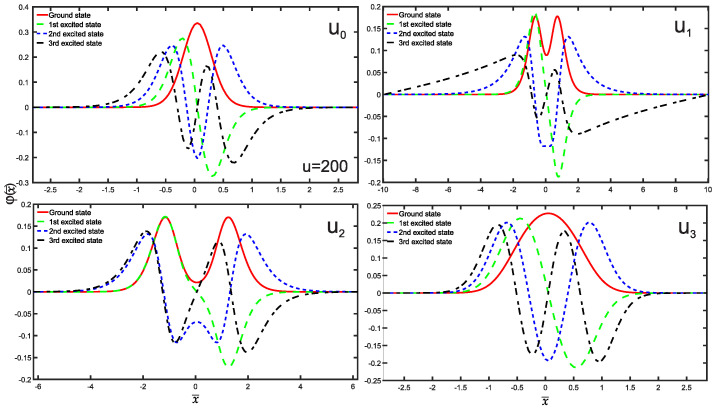
The plots displaying the normalized wave functions as a function of position *x* for both single-well ($U_{0}$ and $U_{3}$) and double-well ($U_{1}$ and $U_{2}$) potentials. The ground state is represented by a solid red line, the 1st excited state by a green dashed line, the 2nd excited state by a blue dotted line, and the 3rd excited state by a black dash-dotted line.

Figure 2 clearly demonstrates that the wave functions corresponding to the single-well potentials $U_{0,3}$ exhibit greater localization compared to those of the double-well potentials $U_{1,2}$. For the sake of clarity, we use distinctive line styles to represent different energy states. The ground state is denoted by a red solid line; the first excited state is represented by a green dashed line; the second excited state is shown as a blue dotted line; the third excited state is depicted as a black dash-dotted line.

Once the wave function is obtained, the Shannon information entropy densities, denoted as $\rho_{s}\left(\bar{x}\right)$ and $\rho_{s}\left(\bar{p}\right)$ ($\bar{p}=L\;p$), can be evaluated as follows [8,9]:

(5) $$\begin{array}{c} \rho_{s}\left(\bar{x}\right)={\left|\varphi \left(\bar{x}\right)\right|}^{2}\ln\left(|\varphi \left(\bar{x}\right){|}^{2}\right),\\ \rho_{s}\left(\bar{p}\right)={\left|\varphi \left(\bar{p}\right)\right|}^{2}\ln\left(|\varphi \left(\bar{p}\right){|}^{2}\right), \end{array}$$

where $|\varphi \left(\bar{x}\right){|}^{2}$ is the probability density function obtained from the squared modulus of the wave function $\varphi (\bar{x})$ and $|\varphi \left(\bar{p}\right){|}^{2}$ is the probability density function obtained from the squared modulus of the wave function $\varphi (\bar{p})$ in momentum space. In general, the wave function in the momentum space $\varphi \left(\bar{p}\right)$ can be calculated by the Fourier transformation

(6) $$\varphi \left(\bar{p}\right)=\frac{1}{\sqrt{2\pi }}\int \varphi \left(\bar{x}\right)e^{-i\bar{p}\;\bar{x}}d\bar{x}.$$

In the current study, however, we employ the Fast Fourier method [45] to numerically compute the wave function in the momentum space as explained above. This also include the numerical integrals required for calculating the Shannon information entropy densities $\rho_{s}\left(\bar{x}\right)$ and $\rho_{s}\left(\bar{p}\right)$ in position and momentum spaces. Despite the numerical approach, these entropy densities remain valuable tools for gaining insights into the information distribution and uncertainty within the system’s position and momentum spaces, respectively. They provide essential information about the localization and spread of information, shedding light on the system’s quantum behavior and properties.

Based on the Shannon information entropy densities for position $\rho_{s}\left(\bar{x}\right)$ and momentum $\rho_{s}\left(\bar{p}\right)$, defined in Equation (5), the Shannon information entropies for position ${\bar{S}}_{x}$ and momentum ${\bar{S}}_{p}$ can be calculated as follows [8,9]:

(7) $$\begin{array}{c} {\bar{S}}_{x}=-\int_{-\infty }^{\infty }\rho_{s}\left(\bar{x}\right)d\bar{x},\\ {\bar{S}}_{p}=-\int_{-\infty }^{\infty }\rho_{s}\left(\bar{p}\right)d\bar{p}, \end{array}$$

which depend on $\bar{u}$ only. That is to say, they are *L*-independent.

These entropy measures provide quantifiable information about the uncertainty and information content in the system’s position and momentum spaces, respectively. They are valuable tools for understanding the overall behavior and characteristics of the quantum system under investigation. In the current choice, the traditional entropies can be represented as

(8) $$S_{x}=\ln L+{\bar{S}}_{x},\;\;\;S_{p}=-\ln L+{\bar{S}}_{p}.$$

For simplicity, we take L=1 in our calculation. Beckner, Bialynicki–Birula, and Mycielski have derived an important uncertainty relation [46,47]. This relation serves as a fundamental result in quantum mechanics and provides insights into the inherent uncertainty and limitations associated with simultaneous measurements of position and momentum. The uncertainty relation can be expressed as:

(9) $$S_{x}+S_{p}\geq D\left(1+\ln\pi \right),$$

where *D* stands for the spatial dimension (D=1 here).

This relation highlights that there is a fundamental limit to how precisely both the position and momentum of a quantum system can be simultaneously determined. Any attempt to reduce the uncertainty in one observable will inevitably lead to an increase in the uncertainty of the other observable. The uncertainty relation plays a crucial role in quantum mechanics and underlines the profound nature of uncertainty at the quantum level.

Before concluding this section, it is important to consider the Fisher information, which was initially recognized for its significance by Sears et al. [48]. They demonstrated that the kinetic energy could be viewed as a measure of information distribution. For a comprehensive understanding of its wide-ranging applications, readers may refer to our recent study [49] for further details. A key distinction between Fisher information and Shannon information lies in their local characteristics. The Fisher entropy exhibits a local nature, setting it apart from Shannon information. Mathematically, the Fisher entropy is defined as an expectation value of the logarithmic gradient of density or as the gradient functional of density. Its explicit definition is given by [50]

(10) $${\bar{F}}_{x}=\int_{a}^{b}\frac{{\left[\rho^{\prime }\left(\bar{x}\right)\right]}^{2}}{\rho (\bar{x})}d\bar{x}=4\int_{a}^{b}{\left[\varphi^{\prime }\left(\bar{x}\right)\right]}^{2}d\bar{x},$$

where the probability density is defined as $\rho \left(\bar{x}\right)={\left|\varphi \left(\bar{x}\right)\right|}^{2}$.

## 3. Results and Discussion

**Figure 3:**
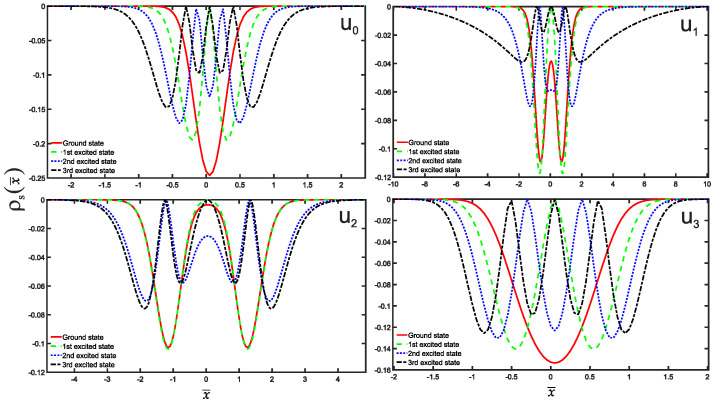
The position entropy density plots as a function of position $\bar{x}$ are presented for potential wells $U_{i}$, where *i* takes values from 0 to 3. Each plot illustrates the position entropy density for different energy states, distinguished by various line styles and colors as above Figure 2.

**Figure 4:**
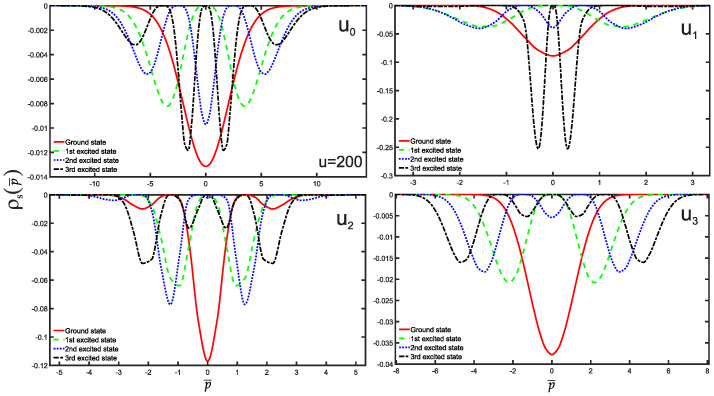
Same as above Figure 3 but for the momentum entropy density case for the potentials $U_{0,1,2,3}$.

We are now in a position to present the results of this study, which include the normalized wave functions (refer to Figure 2), as well as the position and momentum entropies densities, denoted as $\rho_{s}\left(\bar{x}\right)$ and $\rho_{s}\left(\bar{p}\right)$, along with the Shannon entropy. For clarity and simplicity, we will focus on the ground state, the first three excited states, and some higher excited states. The variation of position and momentum entropies densities with respect to the variables $\bar{x}$ and $\bar{p}$, respectively, is shown in Figure 3 and Figure 4. In these figures, the ground state is represented by a solid red line, the first excited state by a green dashed line, the second excited state by a blue dotted line, and the third excited state by a black dash-dotted line. Furthermore, we have made an interesting observation: the position entropy density $\rho_{s}\left(\bar{x}\right)$ for the single-well potentials $U_{0}$ and $U_{3}$ exhibits a more localized behavior compared to that of the double-well potentials $U_{1}$ and $U_{2}$. However, the situation is reversed when it comes to the momentum entropy density $\rho_{s}\left(\bar{p}\right)$. These findings shed light on the intriguing differences between single-well and double-well potentials in terms of localization properties for position and momentum entropies densities.

**Figure 5:**
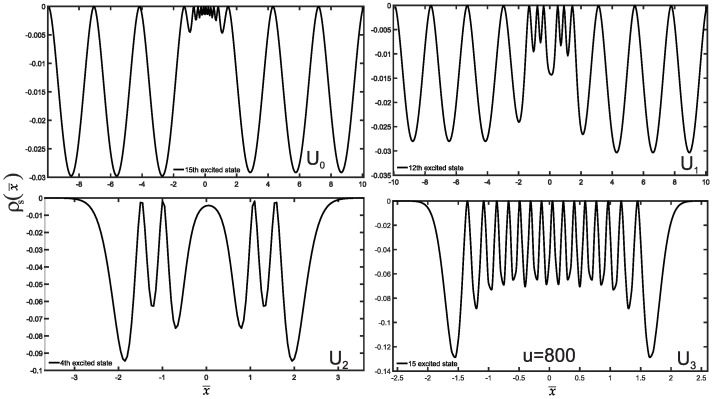
Plots of position entropy density for normalized higher excited states for potential wells. When u=800, we choose the 15th excited state for $U_{0}$ and $U_{3}$ but 12th and 4th excited states for $U_{1}$ and $U_{2}$, respectively.

**Figure 6:**
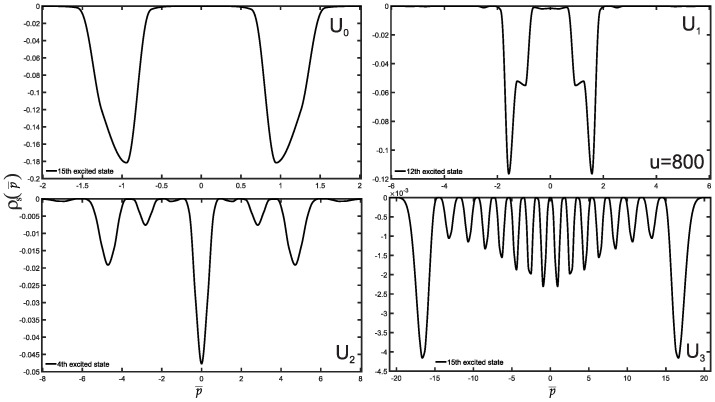
Same as Figure 5 but for the momentum entropy density.

Furthermore, we have performed an investigation into the position and momentum entropies densities of higher excited states. Specifically, we examined the 15th excited state for the single-well potentials $U_{0}$ and $U_{3}$, and the 12th and 4th excited states for the double-well potentials $U_{1}$ and $U_{2}$, respectively, as illustrated in Figure 5 and Figure 6. Our findings reveal a compelling trend: the position entropy density increases farther away from the origin. This is because the particles in the highly excited state want to escape the potential well more.

**Figure 7:**
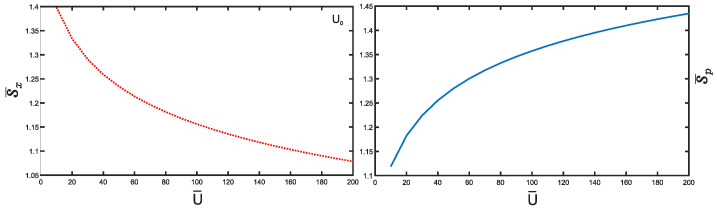
Position and momentum entropy ${\bar{S}}_{x}$ and ${\bar{S}}_{p}$ for potential $U_{0}$. Here, we only consider the ground state.

**Figure 8:**
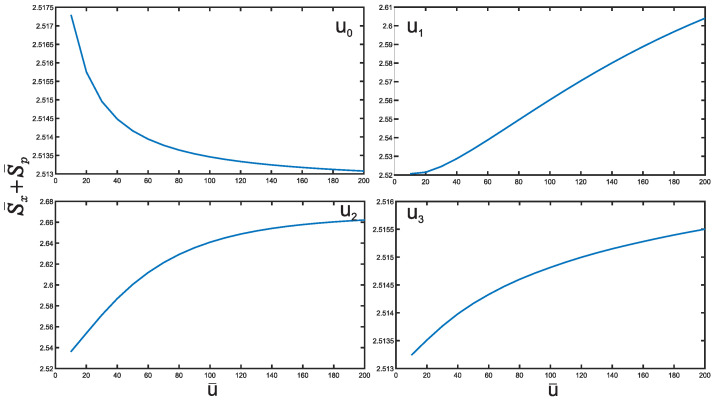
The sum of position and momentum entropies ${\bar{S}}_{x}$ and ${\bar{S}}_{p}$ for hyperbolic potentials.

We now study the Shannon entropy. Figure 7 presents an analysis of the position and momentum entropies, denoted as ${\bar{S}}_{x}$ and ${\bar{S}}_{p}$, respectively, as a function of the depth $\bar{u}$ for the potential well $U_{0}$. For simplicity, we focus on the ground state for all potentials. Notably, we observe that the position entropy ${\bar{S}}_{x}$ decreases with increasing depth $\bar{u}$ of the potential well, while the momentum entropy ${\bar{S}}_{p}$ exhibits the opposite behavior. Similar trends are observed for the other cases $U_{1,2,3}$ mirroring the behavior of $U_{0}$. Let us delve into the BBM inequality and its implications for all potentials $U_{0,1,2,3}$, as demonstrated in Figure 8, where the inequality is consistently satisfied. We notice that the sum ${\bar{S}}_{x}+{\bar{S}}_{p}$ for $U_{0}$ notably decreases as the depth $\bar{u}$ of the potential well increases. Conversely, for the cases $U_{1,2,3}$, this sum increases with depth $\bar{u}$. This suggests that the sum for $U_{0}$ becomes more stable compared to the cases $U_{1,2,3}$ as the depth $\bar{u}$ increases. The reason behind the more restricted movement of particles in the potential well $U_{0}$ compared to $U_{3}$ is simply due to the size difference between the two potential wells. The smaller width of $U_{0}$ confines particles to a more limited region, whereas the broader width of $U_{3}$ allows for a wider range of motion. Additionally, it is observed that the sum of the cases $U_{0}$ and $U_{3}$ remains nearly constant within a relative value of 0.01 as $\bar{u}$ increases, with its value consistently above the minimum value of 2.144. It should be recognized that the decrease of the sum for $U_{0}$ case cannot go below this limit. In particular, at the extremely large $\bar{u}$ the sum just approaches to this value. This implies that the corresponding level becomes more and more Gaussian. This fact can be supported by the Taylor expansions of the corresponding potentials:

(11) $${\bar{U}}_{0}\left(\bar{x}\right)=-1+3{\bar{x}}^{2}-5{\bar{x}}^{4}+\ldots ,\;\;\;\;{\bar{U}}_{3}\left(\bar{x}\right)=-1+{\bar{x}}^{6}-2{\bar{x}}^{8}+\ldots .$$

With the growth of the depth of the potential well $\bar{u}$, the shape of the potential ${\bar{U}}_{0}\left(\bar{x}\right)$ becomes more and more harmonic ($∼{\bar{x}}^{2}$), which leads to the saturation of the Shannon entropy uncertainty relation whereas the potential ${\bar{U}}_{3}\left(\bar{x}\right)$ approaches to a sextic shape ($∼{\bar{x}}^{6}$), which results in the growth of the sum.

**Figure 9:**
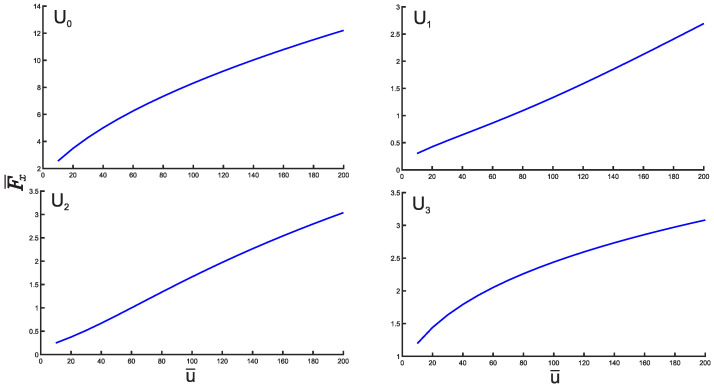
Plots of the Fisher entropy as a function of the depth $\bar{u}$ for the potentials $U_{0,1,2,3}$.

**Figure 10:**
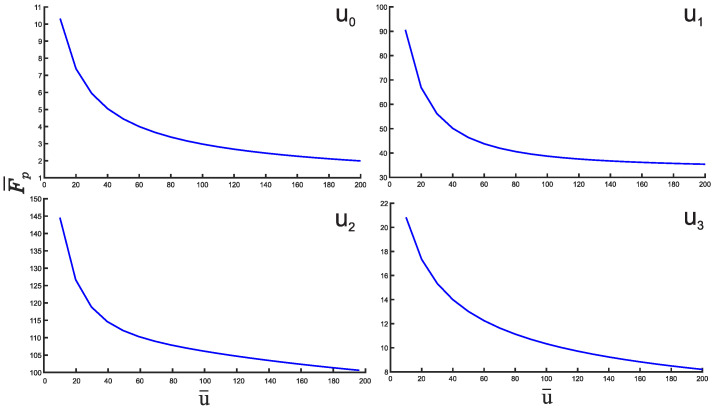
Plots of the Fisher entropy in the momentum space as a function of the depth $\bar{u}$ for the potentials $U_{0,1,2,3}$.

Finally, we present the plots of the Fisher entropy as a function of the depth $\bar{u}$ for all potentials $U_{0,1,2,3}$. Figure 9 clearly shows that the Fisher entropy ${\bar{F}}_{x}$ increases with the increasing depth $\bar{u}$ for all mentioned potentials. For completeness, we also show the Fisher entropy ${\bar{F}}_{p}$ in the momentum space as shown in Figure 10. In particular, for the potential ${\bar{U}}_{0}\left(\bar{x}\right)$ the product ${\bar{F}}_{x}{\bar{F}}_{p}$ will asymptotically approach from above at the increasing $\bar{u}$ the value of 4, which is the harmonic oscillator limit.

## 4. Concluding Remarks

In this study, we investigated a class of recently proposed hyperbolic potentials through studying the time-independent Schrödinger equation. Our main focus was to analyze whether the BBM inequality held for these potentials. Additionally, we explored the various properties such as the wave function, entropy density, Shannon entropy in both position and momentum spaces. We observed that the behavior of position and momentum entropy densities differed for single- and double-well potentials. Specifically, the position entropy density became more localized for single-well potentials, while it became more delocalized for double-well potentials. As one may expect, the momentum entropy density behaved inversely.

A notable finding was that as the depth of the potential wells $\bar{u}$ increased, the position entropy ${\bar{S}}_{x}$ decreased, whereas the momentum entropy ${\bar{S}}_{p}$ increased. This behavior was distinct for single and double well potentials. Furthermore, we demonstrated that the sum of entropies ${\bar{S}}_{x}+{\bar{S}}_{p}$ for single-well potentials $U_{0,3}$ became more stable with increasing depth $\bar{u}$, compared to the double-well potentials $U_{1,2}$. This observation was consistent with the shape of the potential wells, as depicted in Figure 1. The relatively deeper single-well potentials $U_{0,3}$ constrained the particle motion more than the shallower double-well potentials $U_{1,2}$. Lastly, we explored the Fisher entropy and observed that it exhibited an increasing trend with the depth $\bar{u}$ of the potential wells.

In conclusion, our study sheds light on the behavior of hyperbolic potentials within the context of the time-independent Schrödinger equation. We provide valuable insights into the impact of potential well depth on various properties of the system, emphasizing the distinctions between single and double well potentials. These findings contribute to a deeper understanding of the dynamics of particles in hyperbolic potentials and their corresponding entropy behaviors.

## Data Availability

No new data were created.
